# β2-Adrenoceptor Deficiency Results in Increased Calcified Cartilage Thickness and Subchondral Bone Remodeling in Murine Experimental Osteoarthritis

**DOI:** 10.3389/fimmu.2021.801505

**Published:** 2022-01-13

**Authors:** Gundula Rösch, Dominique Muschter, Shahed Taheri, Karima El Bagdadi, Christoph Dorn, Andrea Meurer, Frank Zaucke, Arndt F. Schilling, Susanne Grässel, Rainer H. Straub, Zsuzsa Jenei-Lanzl

**Affiliations:** ^1^ Dr. Rolf M. Schwiete Research Unit for Osteoarthritis, Department of Orthopedics (Friedrichsheim), University Hospital Frankfurt, Goethe University, Frankfurt am Main, Germany; ^2^ Department of Orthopedic Surgery, Experimental Orthopedics, Centre for Medical Biotechnology, University of Regensburg, Regensburg, Germany; ^3^ Department of Trauma Surgery, Orthopedic Surgery and Plastic Surgery, Universitätsmedizin Göttingen, Göttingen, Germany; ^4^ Institute of Pharmacy, University of Regensburg, Regensburg, Germany; ^5^ Laboratory of Experimental Rheumatology and Neuroendocrine Immunology, Department of Internal Medicine, University Hospital Regensburg, Regensburg, Germany

**Keywords:** osteoarthritis, β2-adrenoceptor, subchondral bone, cartilage, synovium, leptin

## Abstract

**Purpose:**

Recent studies demonstrated a contribution of adrenoceptors (ARs) to osteoarthritis (OA) pathogenesis. Several AR subtypes are expressed in joint tissues and the β2-AR subtype seems to play a major role during OA progression. However, the importance of β2-AR has not yet been investigated in knee OA. Therefore, we examined the development of knee OA in β2-AR-deficient (*Adrb2^-/-^*) mice after surgical OA induction.

**Methods:**

OA was induced by destabilization of the medial meniscus (DMM) in male wildtype (WT) and *Adrb2^-/-^* mice. Cartilage degeneration and synovial inflammation were evaluated by histological scoring. Subchondral bone remodeling was analyzed using micro-CT. Osteoblast (alkaline phosphatase - ALP) and osteoclast (cathepsin K - CatK) activity were analyzed by immunostainings. To evaluate β2-AR deficiency-associated effects, body weight, sympathetic tone (splenic norepinephrine (NE) *via* HPLC) and serum leptin levels (ELISA) were determined. Expression of the second major AR, the α2-AR, was analyzed in joint tissues by immunostaining.

**Results:**

WT and *Adrb2^-/-^* DMM mice developed comparable changes in cartilage degeneration and synovial inflammation. *Adrb2^-/-^* DMM mice displayed elevated calcified cartilage and subchondral bone plate thickness as well as increased epiphyseal BV/TV compared to WTs, while there were no significant differences in Sham animals. In the subchondral bone of *Adrb2^-/-^* mice, osteoblasts activity increased and osteoclast activity deceased. *Adrb2^-/-^* mice had significantly higher body weight and fat mass compared to WT mice. Serum leptin levels increased in *Adrb2^-/-^* DMM compared to WT DMM without any difference between the respective Shams. There was no difference in the development of meniscal ossicles and osteophytes or in the subarticular trabecular microstructure between *Adrb2^-/-^* and WT DMM as well as *Adrb2^-/-^* and WT Sham mice. Number of α2-AR-positive cells was lower in *Adrb2^-/-^* than in WT mice in all analyzed tissues and decreased in both *Adrb2^-/-^* and WT over time.

**Conclusion:**

We propose that the increased bone mass in *Adrb2^-/-^* DMM mice was not only due to β2-AR deficiency but to a synergistic effect of OA and elevated leptin concentrations. Taken together, β2-AR plays a major role in OA-related subchondral bone remodeling and is thus an attractive target for the exploration of novel therapeutic avenues.

## Introduction

Osteoarthritis (OA) is the most prevalent chronic degenerative joint disease that affected worldwide 303 million people in 2017 (1). OA pathogenesis involves the whole joint with destruction of cartilage, inflammation of synovium, formation of osteophytes, sclerosis of subchondral bone as well as degeneration of ligaments and menisci. (2). These pathological processes lead to the major symptoms joint stiffness and pain, which result not only in limited movement and reduced quality of life but also in immense socio-economic costs. (3). Although OA has been already known for more than 250 years, the exact underlying complex molecular mechanisms of its progression are still unclear (4). Currently, there is still no causal therapy that effectively addresses joint degeneration and inflammation, only symptomatic treatments exist aiming to reduce pain (5). As a result, joint replacement is often the last effective therapy (6). In order to develop novel therapeutic options, it is essential to better understand the multifactorial pathophysiology of OA.

In recent years, the contribution of neurotransmitters and receptors of the sympathetic nervous system (SNS) to the pathophysiology of OA became more and more evident (7–9). The SNS is responsible for the body’s fight or flight response mediated by the neurotransmitters epinephrine (E) and norepinephrine (NE) among others (10). NE was detected in the synovial fluid of OA patients in physiologically relevant concentrations, whereas E was not measurable (11). NE mediates its effects *via* the adrenergic receptors (ARs), which are subdivided into several groups: α1-AR (α1a, α1b, and α1d), α2-AR (α2a, α2b, and α2c) and β-AR (β1, β2, and β3) (12).

Different α- and β-AR subtypes were detected in various tissues related to OA development such as synovium, cartilage, and subchondral bone (8, 13–16). The effects mediated by these ARs have been investigated with regard to OA in different cell types of the joint *in vitro*. Activated α1-ARs increase both the proliferation and apoptosis of human OA chondrocytes (17) as well as proliferation of osteoblasts and activity of osteoclasts (13). Thus, the net effect of α1-ARs seems to be balanced between anabolic and catabolic influences. In contrast, α2a-ARs act mainly in a catabolic manner by decreasing the synthesis of type II collagen and sulphated glycosaminoglycans (GAGs) in human synovial stem cells undergoing chondrogenesis (18). Similarly, in rat chondrocytes, aggrecan expression decreased and MMP-3 and MMP-13 expression increased after α2-AR activation (19).The catabolic effect of the α2-AR on cartilage was confirmed in a temporomandibular joint (TMJ) OA model in rats (19). Moreover, a similar catabolic effect was observed in the subchondral bone of the TMJ after α2-AR activation (19). The β2-AR mediated mainly anabolic effects in human OA chondrocyte cultures by inhibiting the gene expression of *MMP13* and *IL-8* as well as inducing sGAG and *COL2A1* gene expression (17). Furthermore, in the above-mentioned TMJ OA model, activation of the β2-AR resulted in subchondral bone loss (20). In synovial tissue, targeting α2- or β2-ARs exerts dual effects depending on the neurotransmitter concentration. In high concentrations, NE inhibited TNF as well as IL-8 *via* β2-AR but in low concentrations NE increased TNFα release *via* α2-AR (21, 22). However, at present no study exists so far that investigated the influence of the distinct AR subtypes on synovium, cartilage and bone in the knee joint at the same time in the living organism.

Our intention was to analyze the contribution of one of the major ARs, the β2-AR, to OA progression *in vivo* using β2-AR-deficient (*Adrb2^-/-^*) mice. We hypothesized that the lack of β2-AR results in accelerated articular cartilage degeneration, subchondral bone thickening as well as increased synovial inflammation. In order to test this hypothesis, OA was induced in the knee joints of *Adrb2^-/-^* mice by the destabilization of the medial meniscus and disease progression was analyzed.

## Materials and Methods

### Animals

Ten weeks old male C57BL/6J mice were purchased from Janvier Laboratories (Le Genest St. Isle, France). Male *Adrb2^-/-^* mice of the same age and with C57BL/6J background were kindly provided by the group of Prof. Susanne Grässel (Dept. of Orthopedic Surgery, University of Regensburg, Germany). According to the strict animal welfare rules and regulations (the principles of the 3Rs), we mated homozygous *Adrb2^-/-^* mice for breeding of the needed male *Adrb2^-/-^* mice and male WT animals at the same age were purchased commercially. Thus, no littermates were used in this study. Mice were housed at 5 animals per cage, were allowed to adapt to animal laboratory conditions for 2 weeks, and kept under standard housing conditions on a 12-hour light/dark cycle with unrestricted access to standard food and water. All experiments were approved by and conducted according to institutional and governmental regulations for experimental animal usage (Ethical Review Committee, Government of Unterfranken, 55.2-2532-2-368).

### OA Induction

Mice were assigned into 4 groups: WT Sham, WT DMM, *Adrb2^-/-^* Sham, *Adrb2^-/-^* DMM. On day 0, mice underwent DMM surgery to induce knee OA or Sham surgery as described previously (23). Mice were randomly chosen for DMM or Sham surgeries by a technician (this person performed the necessary preparations such as weighing or shaving of the knees before surgery). Mice were anaesthetized by intraperitoneal injection of ketamine-hydrochloride (90-120 mg/g body weight; Medistar Arzneimittelvertrieb GmbH, Ascheberg, Germany) and xylazine (6-8 mg/g/body weight; Serumwerk Bernburg, Bernburg, Germany). DMM or Sham surgery was performed on the right knee joints. In the Sham groups, the joint capsule was opened without transecting the medial meniscotibial ligament (23). After operation, mice were administered subcutaneous analgesia (buprenorphine in 0.9% NaCl solution, 0.1 mg/g body weight; Buprenovet^®^, Bayer Vital GmbH, Leverkusen, Germany). After 2, 4, 8 and 12 weeks (various termination time points), animals were sacrificed by asphyxiation with CO_2_. Body length, body weight, and spleen weight were determined, and limbs were harvested for histological and for micro-CT examinations. We analyzed 8 animals per group for each time point (2, 4, 8 and 12 weeks; histology and immunostainings, as well as weighing and spleen analysis) except for µCT which was only performed 8 weeks after surgery and therefore 5 additional animals were needed at this time point (n=13 per group for the 8 week time point).

### OARSI and Synovitis Scoring

To evaluate OA severity, a standardized histopathological assessment of cartilage degeneration was applied according to the standards suggested by the Osteoarthritis Research Society International (OARSI) (24). According to this scoring system, 6 sections per mouse at 80 μm intervals were stained with DMMB. Features of synovial inflammation were scored in the same sections in defined regions of interest, modified after Krenn et al. (25). From each mouse, 3 stained sections were selected to assess synovitis features by counting the cells in the stroma and characterizing the lining layer enlargement with a scale from none (0), slight (1) and moderate (2) to strong (3). After summarizing the values of both features, the following classification was obtained: 0-1, no synovitis; 2-3, mild synovitis; 4-6, severe synovitis. OARSI and synovitis scoring was performed blinded with respect to the treatment groups by independent observers. The scores are average scores of 6 sections per animal and averages from the three investigators involved in scoring.

### Micro-CT Measurements

For image acquisition, paraformaldehyde(PFA)-fixed complete knee joints were placed in 70% ethanol and scanned in a micro-CT device (Scanco µCT 50, Brüttisellen, Switzerland) with the following settings: isotropic voxel size = 3.4 µm, source voltage = 90 kVp, intensity = 88 µA, integration time = 1500 ms, projections/180° = 1000, and a 0.5 mm aluminum filter as the attenuating substance for beam hardening. Tomograms were then reconstructed in Scanco’s OpenVMS software for general three-dimensional visualization.

To extract the calcified cartilage (CC) layer from its underlying subchondral bone plate, a semi-automatic segmentation process was implemented, where two initial point clouds were created by setting the thresholds values at 396.0 and 933.0 mg hydroxyapatite (HA)/cm^3^, respectively. This provides an initial rough estimation of the layers, which can be enhanced by an interactive step based on a modified Seeded Region Growing technique (26). The calculated bins of values were then used to create histograms, and subsequent colormaps of the CC thickness in each condyle. Colormaps were scaled to a maximum value of 80 μm for all samples. Moreover, the thickness of the subchondral bone plate was measured by the ImageJ software (version 1.52a, NIH, Bethesda, MD, USA) in three equally-spaced coronal cross-sections of the joint. The intercondylar eminence was excluded from measurement, while the final reported value for each condyle was the mean ± SEM of 60 spots in the three coronal planes.

The medial condyle length was measured as an indicator for the osteophyte formation. This was defined as the distance from the center of the condyle in proximity of the trochlear groove to the medial prominence (27). The bone morphometry indices were calculated in two volumes of interest: (1) a ≈ 0.2 mm^3^ region within the medial epiphysis located between the lower margin of the subchondral bone plate and the epiphyseal line, and (2) a ≈ 1.2 mm^3^ volume of interest (VOI) located 300 μm distally from the epiphyseal line for measurement of the sub-articular trabecular morphometry. For both VOIs, an optimal threshold settings according to Scanco’s OpenVMS software (lower threshold: 685.3 mg HA/cm³, upper threshold: 3000 mg HA/cm³, Gauss Sigma: 0.8, Gauss Support: 1) was implemented, while the manually-drawn contouring excluded the endocortical surface in accordance with standard guidelines (28). To characterize the heterotopic ossification of the meniscus before and after the DMM surgery, the anterior meniscal ossicles were manually contoured and segmented. The VOIs in Sham and DMM mice were ≈ 0.4 mm3 and 1.0 mm^3^, respectively, owing to the post-traumatic irregular surface expansion of the ossicles at the site of the injury. Hence, the bone volume (BV), bone mineral density (BMD), bone surface (BS), and the BS/BV of the anterior meniscus were measured and compared for the WT and β2-AR deficient mice before and after the surgery.

### Histology and Immunohistochemistry of Joint Tissues

Limbs were fixed in 4% PFA [in 1x phosphate-buffered saline (PBS)] overnight, washed in 1x PBS for 24 h, decalcified in 10% Tris-ethylenediaminetetraacetic acid (EDTA) for 10-14 days, and embedded in paraffin in frontal orientation. Serial sections (8 µm) were stained with 1,9-dimethyl-methylene blue (DMMB, Sigma-Aldrich, Munich, Germany) for OARSI (Osteoarthritis Research Society International) (24) and synovitis scorings (25).

The expression of the most relevant AR besides β2-AR, namely the α2A-AR, in region of osteophytes, cartilage and synovium as well as markers of osteoblast (alkaline phosphatase (ALP)) and osteoclast activity (Cathepsin K (CatK)) in the subchondral bone were analyzed by immunostaining. Controls for β2-AR deficiency were performed by β2-AR staining. Sections were blocked using 2.5% normal horse serum blocking solution for 20 min at room temperature (Cat.No: S-2012, Vector Laboratories, Burlingame, USA) followed by incubation with primary antibodies over night at 4°C (α2A-AR – 14266-1-AP 1:100, Proteintech, Rosemont, US; β2-AR - 1:100, PA5-14117, Thermo Fisher, Rockford, USA; CatK – 1:200 PA05-109605, Thermo Fisher Scientific, Waltham US; and ALP - 1:200 AF2910, RnDsystems, Minneapolis, USA). Primary antibodies were detected using ImmPRESS^®^ HRP-Anti-Rabbit and HRP-Anti-Goat IgG (Peroxidase) Polymer Detection Kit (Vector Laboratories, Burlingame, USA).

### Quantification of AR-, ALP-, and CatK-Positive Stainings

In order to determine the number of cells expressing α2A-AR, sections were stained as described above. AR-positive cells were counted in comparable regions as shown in **Supplementary Figure 1** and by independent observers.

ALP and CatK localization were quantified by pixel area count and reported as a percentage of the total tissue area specified (**Supplementary Figure 1**) using the software Image J (NIH Image, Bethesda, USA) (29). ALP- and CatK-positive cells were quantified by determining the positively stained area because due to the cutting plane in tissue sections it is not always clear, whether stained areas originated from one or more cells.

### Determination of Splenic NE Levels

The spleen is richly innervated by sympathetic nerve fibers (30, 31). Therefore, in order to compare the sympathetic activity of WT and *Adrb2^-/-^* mice, the concentration of the NE was measured in spleen homogenates by HPLC with electrochemical detection as previously described by us (32). The collection of the spleens was performed in the morning between 8:00 and 11:00 to avoid variability due to circadian rhythm.

### Serum Leptin Quantification by ELISA

To investigate, whether the effects of β2-AR deficiency on the subchondral bone were mediated by adipose tissue-derived factors, the concentration of the most relevant adipokine leptin (33) was determined in serum samples using Mouse/Rat Leptin Quantikine ELISA Kit (MOB00B, RnDsystems, Minneapolis, USA according to the manufacturer´s protocol. The collection of the serum was performed in the morning between 8:00 and 11:00 to avoid variability due to circadian rhythm.

### Statistical Analysis

**Figure 1:**
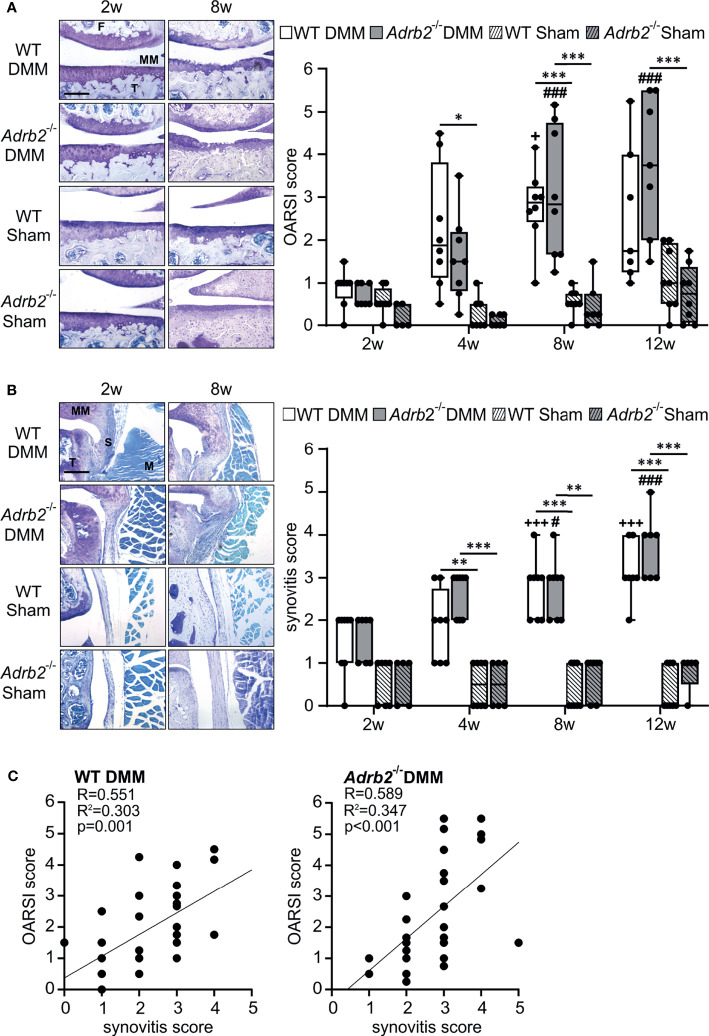
Time course of cartilage degeneration and synovial inflammation following DMM. **(A)** Histological analysis and OARSI scores of the medial tibiofemoral articular cartilage contact area using DMMB staining in WT and *Adrb2^-/-^* mice 2, 4, 8, and 12 weeks after DMM or Sham surgery (bar: 200 µm). p≤0.001 when compared to 2 weeks WT DMM. Data are presented as box plots with whiskers. Each black circle represents an individual mouse (n=5-8 per group). Significant p-values are presented as * p ≤ 0.05, ***p ≤ 0.001 for comparisons between groups at one time point; ^###^p ≤ 0.001 when compared to 2 weeks *Adrb2^-/-^* DMM. No significance differences observed between WT DMM and *Adrb2^-/-^* DMM. **(B)** Histological appearance of the synovial tissue and synovitis score in WT and *Adrb2^-/-^* mice 2, 4, 8, and 12 weeks after DMM or Sham surgery (DMMB staining, T: tibia, S: synovium, MM: medial meniscus, M: muscle; bar: 200 µm). Data are presented as box plots with whiskers. Each black circle represents an individual mouse (n=5-8 per group). Significant p-values are presented as **p ≤ 0.01, ***p ≤ 0.001 for comparisons between groups at one time point; ^+++^p ≤ 0.001 when compared to 2 weeks WT DMM; ^#^p ≤ 0.05, ^###^p ≤ 0.001 when compared to 2 weeks *Adrb2^-/-^* DMM. **(C)** Linear regression of articular cartilage damage (OARSI score) and synovial inflammation (synovitis score) in WT and *Adrb2^-/-^* mice 2, 4, 8, and 12 weeks after DMM surgery.

**Figure 2:**
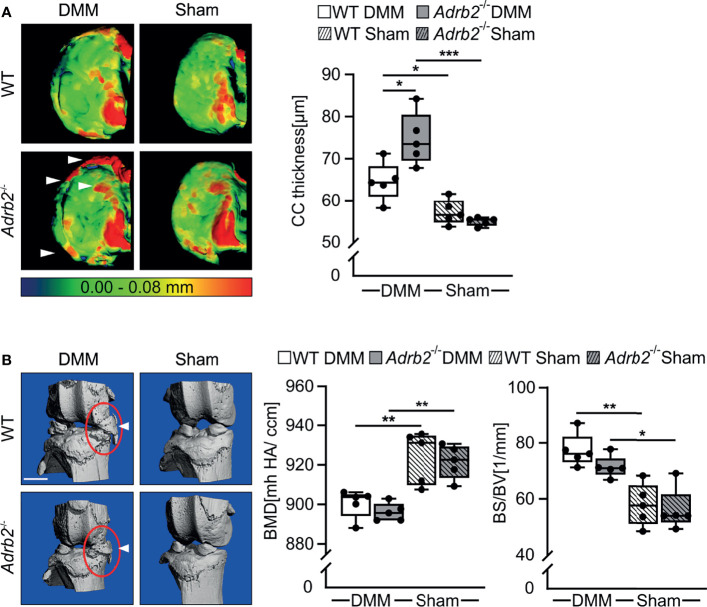
Micro-CT analysis of the calcified cartilage (CC) thickness and meniscal ossicles. **(A)** Representative color-densitometry micro-CT images and quantification of the CC thickness in WT and *Adrb2^-/-^* mice 8 weeks after DMM or Sham surgery. Data are presented as box plots with whiskers. Each black circle represents an individual mouse (n=5 per group). Significant p-values are presented as *p ≤ 0.05, **p ≤ 0.01, ***p ≤ 0.001 for comparisons between groups. **(B)** Representative pictures of meniscal ossicles as well as quantitative differences of their bone specific surface (BS/BV) and bone mineral density (BMD) in WT and *Adrb2^-/-^* mice 8 weeks after DMM or Sham surgery. Data are presented as box plots with whiskers. Each black circle represents an individual mouse (n=5 per group). Significant p-values are presented as *p ≤ 0.05, **p ≤ 0.01, for comparisons between groups.

**Figure 3:**
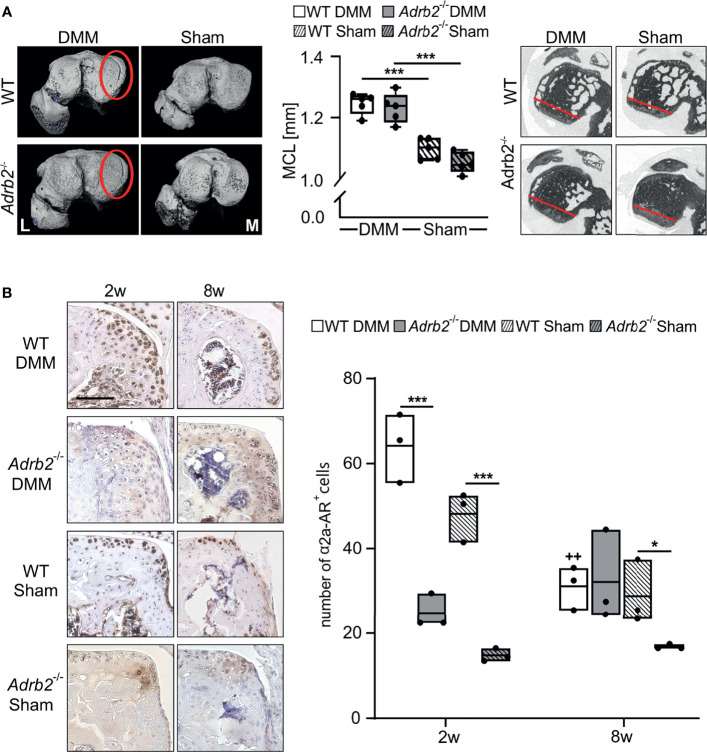
Analysis of the region of osteophyte formation in the medial condyles. **(A)** Representative micro-CT images of the medial condyles with (DMM groups) or without (Sham groups) osteophytes 8 weeks after surgery. The quantification of the medial condyle lengths in WT and *Adrb2^-/-^* mice 8 weeks after surgery and representative micro-CT images display the medial condyle. Data are presented as box plots with whiskers. Each black circle represents an individual mouse (n=5 per group). Significant p-values are presented as ***p ≤ 0.001 for comparisons between groups. **(B)** Immunohistochemical detection and quantification of α2A-AR (dark brown) in the region of osteophyte formation in WT and *Adrb2^-/-^* mice 2 and 8 weeks after DMM or Sham surgery (bar: 100 µm). Nuclei are counterstained with hematoxylin (in dark blue). Data are presented as box plots with whiskers. Each black circle represents an individual mouse (n=3 per group). Significant p-values are presented as *p ≤ 0.05, ***p ≤ 0.001 for comparisons between groups. ^++^p≤0.01 when compared to 2 weeks WT DMM.

**Figure 4:**
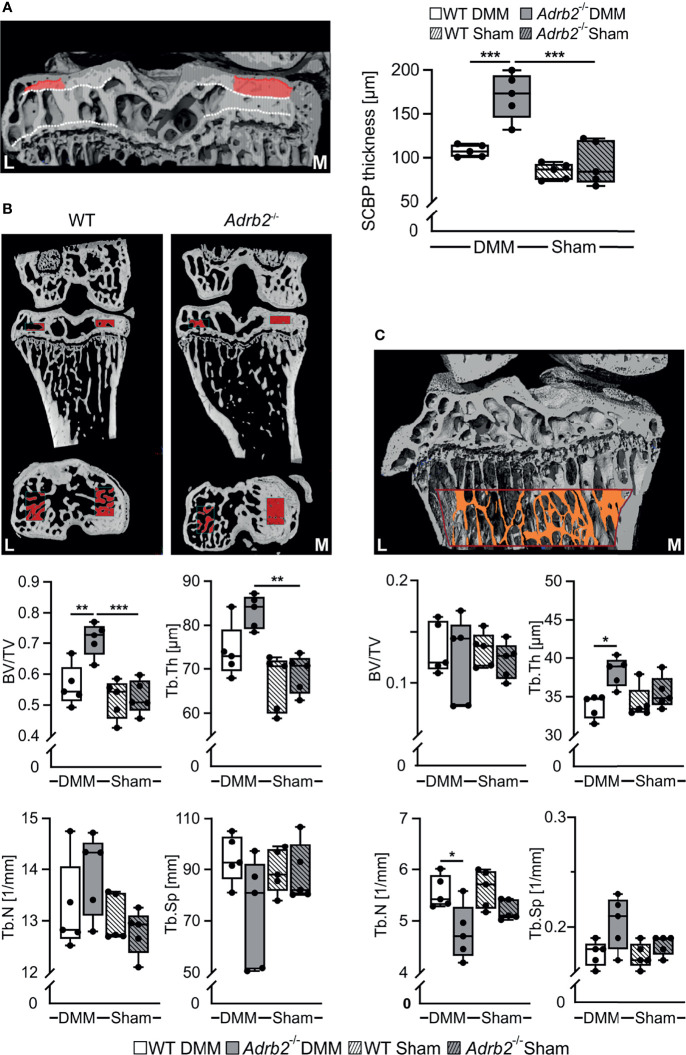
Analysis of the subhcondral bone and subarticular trabecular bone. **(A)** Representative image of subchondral bone plate (SCBP) with the VOI and quantification of the SCBP thickness in WT and *Adrb2^-/-^* mice 8 weeks after DMM or Sham surgery. **(B)** Representative micro-Ct images displaying the volume of interest (VOI, indicated by red rectangles) in the medial epiphysis and quantitative 3D analysis of bone volume to total tissue volume (BV/TV), trabecular spacing (Tb.Sp), trabecular number (Tb.N), and trabecular thickness (Tb.Th) within the VOI in WT and *Adrb2^-/-^* mice 8 weeks after DMM or Sham surgery. **(C)** Representative micro-Ct image displaying the volume of interest (VOI, indicated by red-orange rectangle) in the subarticular trabecular bone and quantitative 3D analysis of bone volume to total tissue volume (BV/TV), trabecular spacing (Tb.Sp), trabecular number (Tb.N), and trabecular thickness (Tb.Th) within the VOI in WT and *Adrb2^-/-^* mice 8 weeks after DMM or Sham surgery. Data are presented as box plots with whiskers. Each black circle represents an individual mouse (n=5 per group). Significant p-values between the treatment groups are presented as *p ≤ 0.05 and **p ≤ 0.01 and ***p ≤ 0.001.

**Figure 5:**
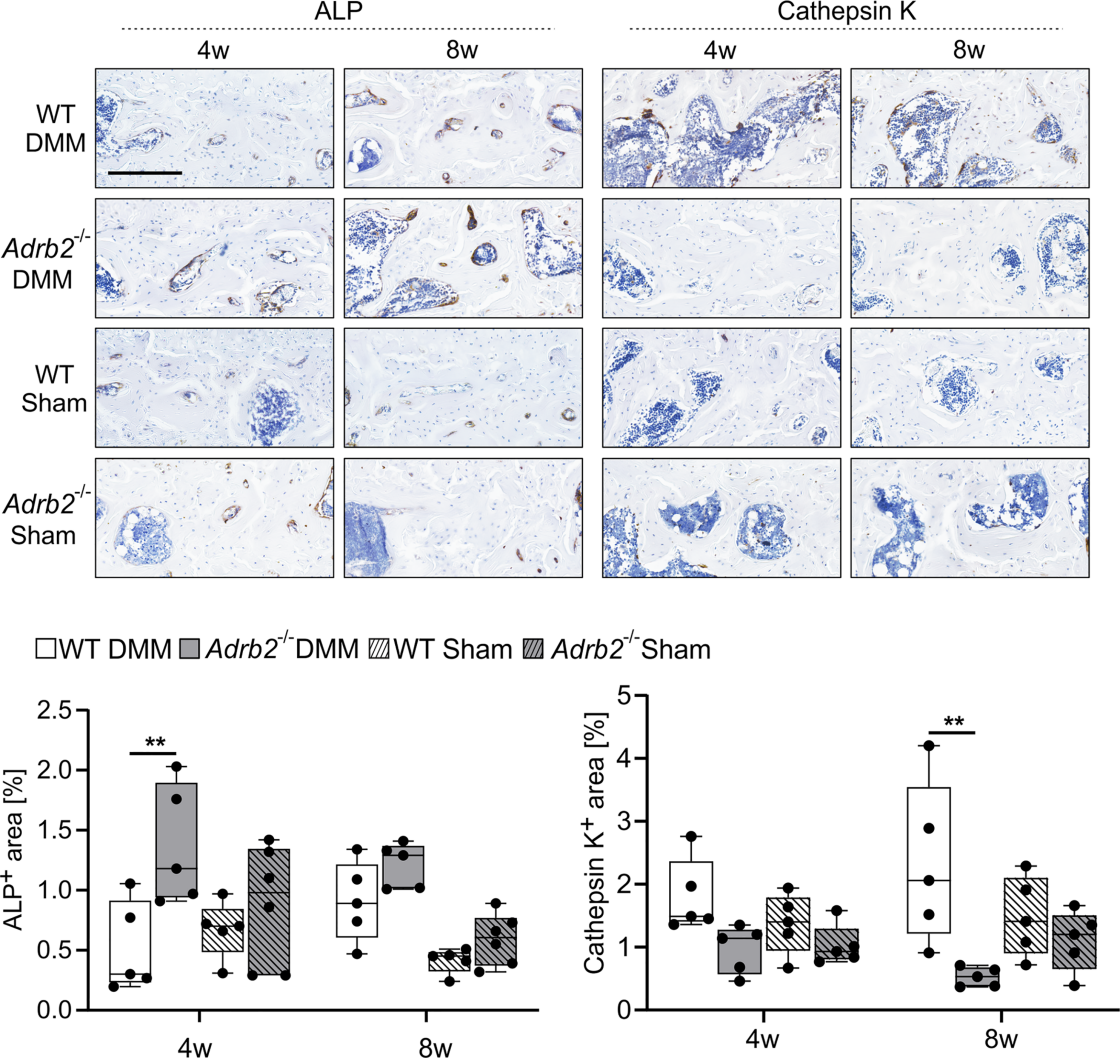
Analysis of the osteoblast and osteoclast activity in the subhcondral bone. Immunohistochemical detection and quantification of ALP (dark brown) and Cathepsin K (dark brown) in the subchondral bone of WT and *Adrb2^-/-^* mice 4 and 8 weeks after DMM or Sham surgery (bar: 200 µm). Nuclei are counterstained with hematoxylin (in dark blue). Data are presented as box plots with whiskers. Each black circle represents an individual mouse (n=5 per group). Significant p-values are presented as **p ≤ 0.01 for comparisons between groups.

**Figure 6:**
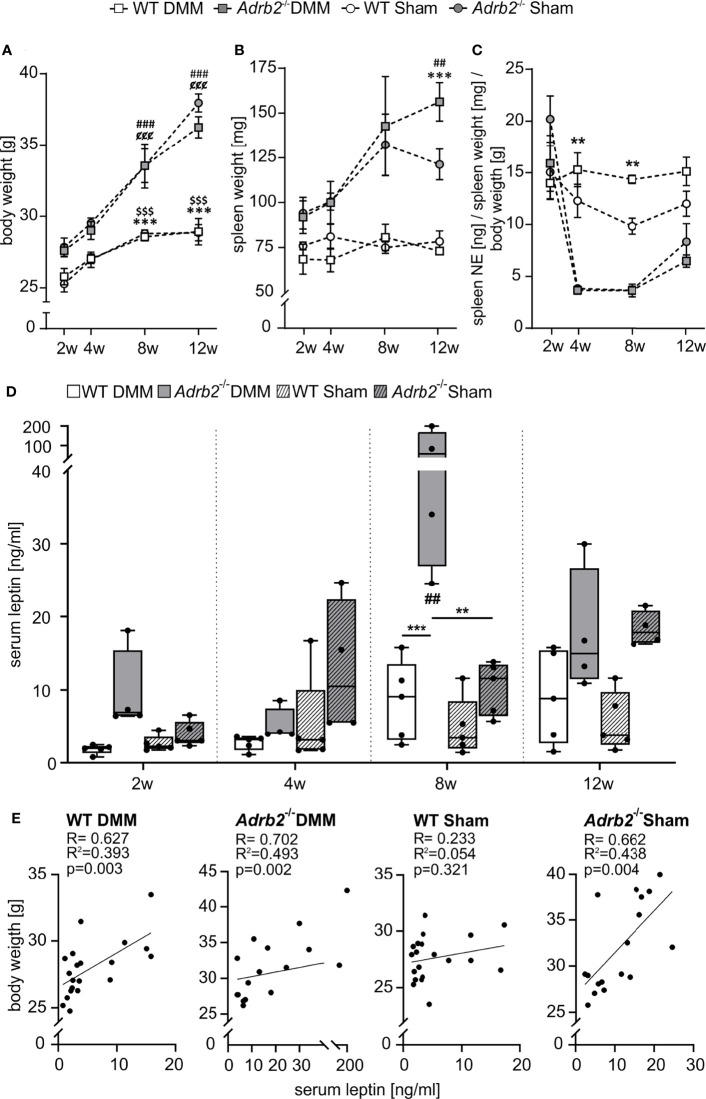
Body weight, spleen weight, splenic NE concentration and serum leptin level. **(A)** Body weight data are represented as means +/- SEM (n=8-10 per group). Significant p-values are presented as ^###^p ≤ 0.001 when compared to 2 weeks *Adrb2^-/-^* DMM; ^$$$^p < 0.001 when compared to 2 weeks WT Sham; ^¢¢¢^p ≤ 0.001 when compared to 2weeks *Adrb2^-/-^* Sham; ***p ≤ 0.001 when WT DMM compared to *Adrb2^-/-^* DMM; ^$$$^p ≤ 0.001 when WT Sham compared to *Adrb2^-/-^* Sham. **(B)** Spleen weight data are represented as means +/- SEM (n=8-10 per group). Significant p-values are presented as ^##^p ≤ 0.01 when compared to 2 weeks *Adrb2^-/-^* DMM; ***p ≤ 0.001 when WT DMM compared to *Adrb2^-/-^* DMM. **(C)** Relative splenic NE concentration data are represented as means +/- SEM (n=3 per group). Significant p-values are presented as **p ≤ 0.01 when WT DMM compared to *Adrb2^-/-^* DMM; ^¢^p ≤ 0.05, ^¢¢¢^p ≤ 0.001 when compared to 2weeks *Adrb2^-/-^* Sham. **(D)** Serum leptin concentration data are presented as box plots with whiskers. Each black circle represents an individual mouse (n=5 per group). Significant p-values are presented as **p ≤ 0.01, ***p ≤ 0.001 for comparisons between groups. **(E)** Linear regression of serum leptin concentrations and body weight in WT and *Adrb2^-/-^* mice 2, 4, 8, and 12 weeks after DMM and Sham surgery.

Experimental group sizes were calculated using the G*Power Software (34). The statistical power of our study was determined based on the readout parameter, cartilage degradation. A minimum of 8 mice per group were needed to observe a minimum 30% difference in means at a power of 80% (σ = 0.2, α = 0.05). For statistical analyses, we used Prism XY (Graph Pad Software, La Jolla, USA). In the text, data are presented as mean ± SEM. p-values of <0.05 were considered significant. For **Figures 1**, **6**, **5** and **Supplementary Figure 5** we performed a three-way ANOVA with the factors surgery, *Adrb2^-/-^* deficiency and time point followed by Bonferroni correction for multiple comparison. Normality was visually checked by the QQ-plot of the residuals (**Supplementary Figure 2**). The data in **Figure 3B** and **Supplementary Figure 4** are not normally distributed. The data were log-transformed and analyzed by a three-way ANOVA with Bonferroni correction for multiple comparison. Normality was visually checked by the QQ-plot of the residuals of the transformed data (**Supplementary Figure 2**). For **Figures 2**, **3A**, **4** we performed a two-way ANOVA with the factors surgery and *Adrb2^-/-^* deficiency, followed by Bonferroni correction for multiple comparison, Normality was visually checked by the QQ-plot of the residuals (**Supplementary Figure S2**). The correlation between OARSI score and synovitis score (**Figure 1C**), as well as serum leptin concentration and body weight (**Figure 6E**) was investigated using linear regression.

## Results

### β2-AR Deficiency Did Not Affect Cartilage Degeneration and Synovial Inflammation

First, β2-AR deficiency was confirmed in healthy *Adrb2^-/-^* mice by immunohistological staining (**Supplementary Figure S3A**).

Then, we analyzed the influence of the β2-AR deficiency on the OA-progression in cartilage and synovium. The progression of cartilage degeneration in the medial tibiofemoral articular cartilage was analyzed by histological staining (**Figure 1A**). As expected, the OARSI score of WT DMM increased (0.875 ± 0.157 to 2.536 ± 0.606) over the observation time from 2 weeks to 12 weeks. Similarly, the OARSI scores in *Adrb2^-/-^* DMM increased (0.714 ± 0.101 to 3.786 ± 0.618) without significant differences compared to WT DMM mice at the respective time points (**Figure 1A**). The OARSI scores of WT and *Adrb2^-/-^* Sham-operated mice were significantly lower compared to the respective DMM mice 4 weeks (only WT p=0.012), 8 weeks (WT p<0.001; *Adrb2^-/-^* p<0.001), and 12 weeks (only *Adrb2^-/-^* p<0.001) after surgery (**Figure 1A**). There was no difference between WT Sham and *Adrb2^-/-^* Sham mice at any time point.

In addition to the OARSI score, synovial inflammation was also determined and revealed a similar pattern as the OARSI score. After DMM surgery, the synovitis scores in WT (1.50 ± 0.267 to 3.143 ± 0.261) as well as *Adrb2^-/-^* animals increased significantly until week 12 (1.571 ± 0.202 to 3.714 ± 0.286) without significant differences between these groups (**Figure 1B**). WT and *Adrb2^-/-^* Sham animals had significantly lower synovitis scores at the 4 week (WT p=0.003; *Adrb2^-/-^* p<0.001), 8 week (WT p<0001; *Adrb2^-/-^* p<0.001) and 12 week (WT p<0001; *Adrb2^-/-^* p<0.001) time points compared to the respective DMM animals (**Figure 1B**). There was no difference between the Sham groups at any time point.

Accordingly, there was a positive correlation between OARSI scores and synovitis scores in both WT DMM (R=0.551, R^2 =^ 0.303, p=0.001) and *Adrb2^-/-^* DMM mice (R=0.589, R^2 =^ 0.347, p<0.001) (**Figure 1C**). As expected, in Sham-operated animals, no association was detected, neither in WT- nor in *Adrb2^-/-^* mice (data not shown).

Furthermore, the number of cells expressing the α2a-ARs was examined 2 and 8 weeks after surgeries to investigate, whether β2-AR deficiency resulted in any compensatory changes in the expression in the medial tibial cartilage and synovial tissue. In the cartilage, no difference between *Adrb2^-/-^* and WT DMM mice was detectable at any time point, while α2a-AR numbers were significantly higher in both *Adrb2^-/-^* and WT Sham mice compared to the respective DMM groups (2w WT p=0.019; *Adrb2^-/-^* p=0.049; 8w WT p<0.001; *Adrb2^-/-^* p<0.001). In contrast, significantly less α2a-AR-positive cells were counted in the synovial tissue of *Adrb2^-/-^* DMM mice than in WT mice (p=0.042). At the 2 week time point, no differences could be detected between DMM and Sham animals. However, 8 weeks after surgery, significantly higher α2a-AR numbers were counted in the synovium of *Adrb2^-/-^* DMM mice compared to *Adrb2^-/-^* Sham mice (**Supplementary Figure 4**, p=0.003).

### Increased Calcified Cartilage Thickness in *Adrb2^-/-^* DMM Mice

Micro-CT analyses of calcified cartilage (CC) thickness revealed that DMM surgery led to a thicker CC in both WT (p=0.041) and in *Adrb2^-/-^* (p<0.001) mice compared to the respective Sham animals as expected (**Figure 2A**). In *Adrb2^-/-^* DMM animals, the thickness of CC was significantly higher compared to the WT DMM mice (**Figure 2A**, p=0.010).

In addition, meniscal ossicles formation in DMM mice was markedly pronounced compared to the Sham groups (**Figure 2B**), although all experimental groups developed ectopic bone in the menisci at the injury site. Both DMM groups had decreased bone mineral density (BMD) (WT p=0.004; *Adrb2^-/-^* p=0.002), as well as a significantly higher bone specific surface (BS/BV) (WT p=0.001; *Adrb2^-/-^* p=0.010) in the meniscal ossicles compared to the respective Sham groups.

### β2-AR Deficiency Did Not Affect Osteophyte Formation But Reduced the Number of α2A-AR-Expressing Cells in This Region

Osteophytes were detected in all WT DMM and *Adrb2^-/-^* DMM mice (5 osteophytes per 5 analyzed mice), while Sham animals did not develop osteophytes (0 osteophytes in 5 examined samples). Osteophyte development in both WT DMM and *Adrb2^-/-^* DMM mice resulted in a significantly increased medial condyle length (MCL) compared to the respective Sham-operated mice (WT p<0.001; *Adrb2^-/-^* p<0.001). No difference in MCL between WT Sham and *Adrb2^-/-^* Sham animals were detected (**Figure 3A**).

The number of α2a-AR-positive cells was examined post-operatively at 2 and 8 weeks. The number of α2a-AR-expressing cells was higher in WT DMM mice 2 weeks after surgery compared to *Adrb2^-/-^* DMM mice (p<0.001) in the region of osteophyte formation. Similarly, in WT Sham compared to *Adrb2^-/-^* Sham mice (p<0.001) (**Figure 3B**). The number of α2a-AR-positive cells in both WT DMM and WT Sham was significantly lower 8 weeks compared to the 2 week time point (**Figure 3B**, p=0.004). Though, after 8 weeks, no significant differences between WT and *Adrb2^-/-^* after Sham or DMM surgery were detectable.

### Increased Subchondral Bone Thickness in *Adrb2^-/-^* DMM Mice

We used micro-CT to investigate the effect of β2-AR deficiency on the subchondral bone as well as the subarticular trabecular bone in the tibiae of WT and *Adrb2^-/-^* animals. First, we examined the changes in the thickness of the subchondral bone plate (SCBP). *Adrb2^-/-^* DMM animals exhibited a significantly thicker SCBP in the medial condyle of the tibia (p<0.001) when compared to WT DMM mice, while there was no increase in *Adrb2^-/-^* Sham animals compared to WT Sham animals (**Figure 4A**). Moreover, *Adrb2^-/-^* DMM mice had significantly increased BV/TV (p=0.006), but no significant differences in Tb.Th, Tb.N and Tb.Sp compared to WT DMM mice 8 weeks after surgery (**Figure 4B**). No differences in any of the parameters were found between *Adrb2^-/-^* Sham and WT Sham animals.

In order to better assess the influence of β2-AR and/or DMM on bone, we also examined the above-described parameters in the subarticular trabecular bone (**Figure 4C**). DMM surgery in *Adrb2^-/-^* mice significantly decreased Tb.Th (p=0.012) and Tb.N (p=0.026) compared to WT DMM mice, while there were no significant changes in BV/TV and in Tb.Sp. (**Figure 4C**). In addition, no significant differences in any of the parameters were detected between *Adrb2^-/-^* and WT Sham animals.

### β2-AR Deficiency Resulted in Increased Osteoblast But Decreased Osteoclast Activity

We analyzed the activity of osteoblasts (ALP) and osteoclasts (CatK) to understand, which processes at the cellular level are responsible for the changes in the subchondral bone (**Figure 5**). *Adrb2^-/-^* DMM animals had a larger ALP positive area by trend compared to WT DMM mice 4 weeks after OA induction. There was no significant difference between the Sham groups at this time point. In contrast, after 8 weeks, the ALP positive area in the medial subchondral bone was unaffected by genotype (WT or *Adrb2^-/-^*) or surgery (DMM or Sham) (**Figure 5**).

Regarding the CatK positive area, the opposite was observed. After 4 weeks, there was no significant difference between WT and *Adrb2^-/-^* DMM, as well as WT Sham and *Adrb2^-/-^* Sham animals. However, 8 weeks after surgery, CatK positive area was significantly higher in WT DMM mice than in *Adrb2^-/-^* DMM mice (**Figure 5**, p=0.002).

### Increased Body and Spleen Weight as Well as Decreased Sympathetic Tone in *Adrb2^-/-^* Mice

To further assess whether there might be relations between body mass and bone structure, changes of body weight were analyzed (**Figure 6A**). There was a strong body weight increase in *Adrb2^-/-^* mice and a less pronounced increase in WT over time. A significant difference between WT and *Adrb2^-/-^* mice appeared 8 weeks (p<0.001) and 12 weeks after DMM (p<0.001), as well as 8 weeks (p=0.001) and 12 weeks (p<0.001) after Sham surgery (**Figure 6A**).

In addition to the increased body weight, we also observed an increase in spleen weight in *Adrb2^-/-^* mice 12 weeks after DMM and Sham (p=0.001) compared to the respective WT animals (**Figure 6B**).

To analyse the influence of β2-AR deficiency on the sympathetic tone, we examined the concentration of splenic NE (**Figure 6C** and **Supplementary Figure 5C**). Regarding absolute splenic NE concentrations, there were no significant differences between WT and *Adrb2^-/-^* mice, except after 2 weeks between *Adrb2^-/-^* Sham and WT Sham (**Supplementary Figure 5C**, p=0.017). However, when splenic NE was normalized to spleen and body weights, both *Adrb2^-/-^* DMM and Sham mice had significantly lower NE levels 4, and 8 weeks after surgery compared to the respective WT groups (**Figure 6C**, 4w p=0.002; 8w p=0.007).

Additionally, we examined the body weights of non-operated control WT and non-operated control *Adrb2^-/-^* mice at the respective time points of surgery. The body weights of both WT and *Adrb2^-/-^* mice increased over time, however, at 12 week time point, *Adrb2^-/-^* mice exhibited significantly higher body weights than WT mice (p<0.001, **Supplementary Figure 5A**). In order to figure out, whether increased body weight is associated with changes in size of the mice, body length was also determined. No significant differences between WT and *Adrb2^-/-^* mice were detected at any time point (**Supplementary Figure 5B**). However, we observed that *Adrb2^-/-^* mice developed markedly higher abdominal fat mass compared to WT mice (**Supplementary Figure S5D**). To the attentive and critical observer, no obvious differences in behaviour and activity were detected.

### Serum Leptin Levels in *Adrb2^-/-^* DMM Mice

In order to examine, whether the effects on the subchondral bone were mediated by adipose tissue-derived factors, the concentration of the leptin in serum samples was measured. β2-AR deficiency led to a significant increase of serum leptin concentrations 8 weeks after DMM compared to the *Adrb2^-/-^* Sham mice (p=0.009) (**Figure 6D**) as well as compared to WT DMM mice (p<0.001) (**Figure 6D**). There was no difference regarding serum leptin levels between WT DMM and WT sham animals. Moreover, there was a strong positive correlation between body weight and blood leptin concentrations in WT DMM mice (R=0.627, R^2 =^ 0.393, p=0.003), in *Adrb2^-/-^* DMM mice (R=0.702, R^2 =^ 0.493, p=0.002), as well as in *Adrb2^-/-^* Sham mice (R=0.662, R^2 =^ 0.438, p=0.003), while there was no association in WT Sham mice (R=0.233; R^2 =^ 0.054, p=0.321) (**Figure 6E**).

## Discussion

Various β2-AR-mediated effects of the SNS on chondrocytes, synoviocytes or cells of the subchondral bone regarding OA pathogenesis have been described by recent studies (9). Most of these studies were performed *in vitro* in monocultures (13, 17, 18), only one investigated the TMJ *in vivo* (20). Therefore, the overall role of β2-AR during OA progression in all involved joint tissues at the same time is still unclear, either in animal or in human OA. The present study analyzed cartilage degeneration, synovial inflammation and subchondral bone remodeling in *Adrb2^-/-^* mice. We demonstrated that although β2-AR deficiency had no influence on articular cartilage degradation and OA-related synovitis, the thickness of calcified cartilage and OA-related subchondral bone changes were aggravated compared to the respective WT control animals.

In order to explore the effects of β2-AR deficiency on OA progression, experimental OA was induced by DMM and at first, cartilage degeneration was evaluated using the OARSI scoring system. Surprisingly, we observed no differences between *Adrb2^-/-^* DMM and WT DMM mice at any time point, although *in vitro* studies performed in human OA chondrocyte culture reported that β2-AR activation resulted predominantly in anabolic effects (17), while in contrast to that, in healthy murine chondrocytes, β2-AR mediated mainly catabolic effects (35). In fact, we expected that in the absence of β2-AR, the above-described catabolic effects of α2-AR should be dominant. However, articular cartilage tissue of *Adrb2^-/-^* mice did not show a higher degree of degeneration. One reason for this phenomenon might be the lower expression of α2-AR in the cartilage of *Adrb2^-/-^* mice, although most existing studies investigating murine AR knockout models rather described that the deficiency of one AR subtype is not compensated by the up- or downregulation of other AR subtypes (36–39).

Since the synovial tissue is one major contributor to OA pathogenesis, the influence of β2-AR deficiency on synovitis progression was also analyzed. The synovial inflammation was progressive with continuously increasing synovitis scores in WT DMM animals as already described in previous studies (40). Interestingly, no differences were detected at any time point between *Adrb2^-/-^* DMM and WT DMM mice. This result was quite surprising because β2-AR activation led to clear anti-inflammatory, while α2-AR activation to pro-inflammatory effects in synovial cell cultures of OA patients (21, 22). Therefore, we expected that β2-AR deficiency results in enhanced synovitis scores due to dominant pro-inflammatory effects mediated by α2-AR. Similar to the situation in articular cartilage, lower numbers of α2-AR were detected in the synovial tissue of both DMM and Sham *Adrb2^-/-^* mice compared to the respective WT mice 2 weeks after surgery. This effect disappeared until week 8 because *Adrb2^-/-^* mice upregulated the synovial expression of α2-ARs, while α2-AR numbers did not change over time. A similar phenomenon was never described before. Therefore, we speculate, that the cells being deficient for one AR subtype try to compensate the lacking effects by regulating other AR subtypes (41). The reason, why *Adrb2^-/-^* mice did not exhibit higher synovitis scores might be that the inflammation is already at its maximum.

We observed an increased thickness of calcified cartilage in WT DMM compared to WT Sham animals knowing that calcification at the interface of cartilage and subchondral bone is clearly associated with cartilage degeneration during OA progression (42). Although the uncalcified zones of articular cartilage were not stronger degenerated in β2-AR-deficienct animals, the microCT analyses revealed that the thickness of calcified cartilage increased in *Adrb2^-/-^* DMM compared to WT DMM mice. There are no existing studies at present describing any effect of the β2-AR on the process of cartilage calcification but in a rat OA model of the TMJ, the α2-AR has been shown to mediate the induction of calcification (19). However, the expression of α2-AR was not higher in the hypertrophic zone of *Adrb2^-/-^* mice suggesting that α2-AR is not involved in this process. Therefore, it is likely that due to the opposing intracellular signaling pathways of α2-AR and β2-AR, the β2-AR acts as a functional antagonist to the α2-AR (10). This seems to be a good reason why *Adrb2^-/-^* DMM mice have increased calcified cartilage thickness.

Further OA-associated events such as increased subchondral bone plate thickness as well as elevated epiphyseal bone volume (43) were detected in the absence of β2-ARs. One possible explanation would be that bone formation increases in *Adrb2^-/-^* DMM mice (44). In fact, the number or activity of osteoblasts increased in these mice indicated by increased ALP-positive regions already 4 weeks post-surgery. This confirms earlier studies demonstrating that the non-selective beta blocker propranolol increased the bone mass in mice and rats (45, 46). In contrast, the non-selective beta-agonist isoproterenol or the β2AR selective agonists clenbuterol or salbutamol decreased the bone mass in mice (46, 47). Another reason for increased bone mass in *Adrb2^-/-^* DMM mice could be reduced bone resorption (44) due to decreased osteoclast activity indicated by less CatK staining after 8 weeks. A specific β2-AR activation has been described to increase rat osteoclast differentiation and activity *in vitro* (20, 48) suggesting that β2-AR-deficiency might be related to suppressed osteoclast differentiation. Other studies reported a significantly increased bone volume in vertebrae and long bones in unchallenged β2-AR knockout mice compared to the respective WT animals supporting our data (49). We believe that the time-dependent differences in osteoblast and osteoclast activities are a result of the differential response of these cells to leptin. We assume that leptin first increases osteoblast proliferation and activity (50), then, osteoblasts secrete factors (OPG or IL-6 among others) that in turn activate osteoclastogenesis and activity (50, 51) (see explanations to leptin below).

Weight gain was faster in *Adrb2^-/-^* mice, although body size did not increase. This was unexpected because an earlier study by Pierroz et al. comparing bone phenotypes in *Adrb2^-/-^* mice did not detect any differences in body weight gain in 4 months old animals when compared to WT (52). As this study was terminated after 4 months, no information on body weight gain in *Adrb2^-/-^* mice at higher ages exist. We detected that an increased body fat mass tissue volume was responsible for the elevated body weight of *Adrb2^-/-^* mice. Furthermore, one study in OA patients described that the expression of the *ADRB2* gene was significantly lower in the adipose tissue of obese patients than in tissues of normal-weight individuals (53). It is known that α- and β-ARs play an important an opposing role in lipolysis. α-ARs, especially the α2-AR, inhibit lipolysis, while β-ARs elicit lipolysis (54). Therefore, the major reason for the increased body fat mass in *Adrb2^-/-^* mice is on the one hand the lack of β2-AR-mediated lipolysis and the other hand the still existing fat deposition by α2-ARs (54, 55). Therefore, besides direct effects of β2-AR-deficiency, elevated subchondral bone mass might be the result of the increased body mass and accordingly higher mechanical loading of the knee joints in *Adrb2^-/-^* mice (56). However, the described subchondral bone effects were not observed in *Adrb2^-/-^* Sham mice.

In addition, elevated serum leptin concentrations of *Adrb2^-/-^* mice might also be responsible for the thickening of the subchondral bone. Although previous studies by the Elefteriou and Karsenty groups demonstrated that increased serum leptin concentrations dramatically reduced bone mass in the vertebral body of mice by inhibiting osteoblast activity and enhancing osteoclast activity (57, 58), other and more recent studies observed the opposite. For example, obese Ob/Ob mice being unable to produce leptin have a reduced bone mass (59). Similarly, leptin treatment of rats resulted in new bone formation, higher bone density and reduction in fracture risk by increasing the proliferation of osteoblasts and inhibiting osteoclastogenesis (60). And finally, in human osteoblasts derived from OA patients, leptin stimulated the proliferation and increased the levels of ALP and type I collagen (61). This fits to the most recent findings demonstrating that adipose tissue plays a critical role in the pathophysiology of OA. The authors described that leptin-mediated effects, rather than body weight, play a predominant role in joint degeneration (62). Interestingly, leptin levels were only elevated in *Adrb2^-/-^* DMM but not in *Adrb2^-/-^* Sham mice, although body weight and fat mass were not different. The reason for this phenomenon is most likely the OA-associated presence of proinflammatory cytokines in *Adrb2^-/-^* DMM mice in contrast to the respective Sham animals. Recent studies investigating obese mice as well as humans described that IL-1β, the major proinflammatory cytokine during OA progression (63), induced the release of leptin from the adipose tissue (64, 65). Thus, the increased bone mass might be a synergistic effect of β2-AR deficiency (directly), β2-AR deficiency-associated increased body fat mass (reduced lipolysis) and serum leptin concentration (indirectly) as well as OA induction itself.

Interestingly, the *Adrb2^-/-^* mice in our study had enlarged spleens. The mouse repository https://www.mousephenotype.org/data/genes/MGI:87938#phenotypesTab described the same phenomenon but without specifying the reason for it. However, one recent study demonstrated that peripheral sympathectomy in mice with type II collagen-induced arthritis led to the retention of immune cells in the spleen (66). Such an immune cell retention in secondary lymphoid organs is mediated by the β2-AR (67, 68). Therefore, increased spleen weight might be the reason of β2-AR deficiency-dependent immune cell accumulation in the spleens of *Adrb2^-/-^* mice.

Since the spleen is richly innervated by sympathetic nerve fibers (30, 31), sympathetic activity can be determined by measuring splenic NE levels Relative NE levels were markedly lower in both *Adrb2^-/-^* DMM and *Adrb2^-/-^* Sham mice compared to the respective WT groups suggesting that β2-AR deficiency resulted in a suppressed peripheral sympathetic activity. This result is not surprising, since previous studies described that the β2-AR induces the presynaptic NE release (69, 70). In addition, DMM itself led to an increased sympathetic activity in WT animals indicated by elevated splenic NE levels, which is most obviously due to SNS activation by OA-related local inflammation. This additional activation of the SNS by DMM can induce the release of NE in secondary lymphoid organs such as the spleen, as already shown in humans with rheumatoid arthritis (10).

Moreover, it has to kept in mind that only male mice were investigated in this study because DMM is a male-dominant model (71). Thus, estrous cyclicity, which would also increase variability and accordingly experimental group sizes (72), can be excluded.

In conclusion, this study demonstrated that β2-AR deficiency might contribute to OA progression by aggravating OA-related calcification at the interface of cartilage and subchondral bone and subchondral bone remodelling, while articular cartilage surface degeneration and synovial inflammation remained unaffected. Although OA is a disease of the whole joint, not all tissues were affected by β2-AR deficiency. According to the saying “Which came first, the chicken or the egg?”, it seems that effects in the calcified layer of cartilage and in the subchondral bone are the driving forces in our model. In fact, it was observed most recently that “subchondral bone changes might occur at the same time as (and possibly earlier than) cartilage changes” (43). We believe that the degeneration of articular cartilage in *Adrb2^-/-^* mice would be more severe at later time points as included into our observation. Thus, our chosen time course might be a limitation of the standard DMM model this study. The thickening of calcified cartilage and subchondral bone in *Adrb2^-/-^* mice is on the one hand directly β2-AR-dependent and mediated by increasing osteoblast and suppressed osteoclast activities. On the other hand, β2-AR deficiency resulted in increased body weight and fat deposition and accordingly in elevated serum leptin levels. Both high serum leptin concentration due to increased body fat mass and the elevated leptin release caused by OA-associated inflammation aggravate the OA-related thickening of subchondral bone in the same way by increasing osteoblast and inhibiting osteoclast activities. Another limitation of the present study is that the contribution of other AR subtypes, except for some work on the α2-AR, was not considered. The present findings clearly demonstrated a major role of β2-AR in OA-related cartilage calcification and subchondral bone changes. Therefore, targeting the β2-AR might represent a novel future treatment option, which might help to develop tissue-specific therapeutic drugs for the prevention of pathological subchondral bone remodeling in OA patients.

## Data Availability Statement

The original contributions presented in the study are included in the article/**Supplementary Material**. Further inquiries can be directed to the corresponding author.

## Ethics Statement

The animal study was reviewed and approved by Ethical Review Committee, Government of Unterfranken, 55.2-2532-2-368. Written informed consent was obtained from the owners for the participation of their animals in this study.

## Author Contributions

ZJ-L and RS contributed to conception and design of the study. DM, ST, CD, AM, FZ, AS, and SG provided study tools and techniques. GR, DM, ST, KEB, CD, and ZJ-L generated the data. GR, ST, KEB, and ZJ-L generated draft figures. GR, ST, and KEB organized the database. GR and ST performed the statistical analysis. GR and ZJ-L wrote the first draft of the manuscript. ST wrote sections of the manuscript. All authors contributed to manuscript revision, read, and approved the submitted version.

## Funding

This study was supported by grants of the Deutsche Forschungsgemeinschaft (to ZJ-L and RS (JE 642/4-1, JE 642/4-2; project number 277277765), to SG (GR 1301/19-2; project number 277277765), and to AS (SCHI 857/9-1, project number 277277765) within the DFG Research Unit FOR2407 ExCarBon, and to FZ (ZA 561/3-1; project number 407168728) within the DFG Research Unit FOR2722).

## Conflict of Interest

The authors declare that the research was conducted in the absence of any commercial or financial relationships that could be construed as a potential conflict of interest.

## Publisher’s Note

All claims expressed in this article are solely those of the authors and do not necessarily represent those of their affiliated organizations, or those of the publisher, the editors and the reviewers. Any product that may be evaluated in this article, or claim that may be made by its manufacturer, is not guaranteed or endorsed by the publisher.

## Supplementary Material

Supplementary Figure 1 Areas of interests in the right murine knee joint. **(A)** Areas of interest for the analyses indicated by red rectangles for articular cartilage (I), synovium (II), subhcondral bone (III) and region of osteophytes (IV). DMMB staining (bar: 500 µm).

Supplementary Figure 2 Quantile-quantile (QQ) plots of residuals present the theoretical quantiles predicted to check the normal distribution for ANOVA.

Supplementary Figure 3 Confirmation of β2-AR deficiency and controls for the immunohistochemical stainings **(A)** Staining of knee joints of β2-AR in WT and Abdrb2*^-/-^* mice with a magnification of 10x for the whole joint (bar: 1,5 mm) and a magnification of 20x for the region of osteophyte formation (bar: 100 µm, F – femur, T – tibia, M - meniscus). **(B)** Negative and isotype controls for the immunohistochemical stainings in the region of osteophyte formation (bar: 100µm).

Supplementary Figure 4 AR expression in the articular cartilage and in the synovium. **(A)** Immunohistochemical detection and quantification of α2A- and β2-AR (dark brown) in the articular cartilage in the medial tibia plateau of WT and *Adrb2^-/-^* mice 2 and 8 weeks after DMM or Sham surgery (bar: 100 µm) and **(B)** in the medial synovium of WT and *Adrb2^-/-^* mice 2 and 8 weeks after DMM or Sham surgery (bar: 200 µm) Nuclei are counterstained with hematoxylin (dark blue). Data are presented as box plots with whiskers. Each black circle represents an individual mouse (n=3 per group). Significant p-values are presented as *p ≤ 0.05, **p ≤ 0.01, ***p ≤ 0.001 for comparisons between groups at one time point. +++p ≤ 0.001 when compared to 4 weeks WT DMM; ## p ≤ 0.01 when compared to 4 weeks *Adrb2^-/-^* DMM.

Supplementary Figure 5 Body weight, body length at the same time points in healthy non-operated WT and *Adrb2^-/-^* mice like 2, 4, 8 and 12 weeks after DMM or Sham surgery in WT and *Adrb2^-/-^* DMM mice. **(A)** Body weight data are represented as means +/- SEM (n=5 per group). Significant p-values are presented as ***p ≤ 0.001, when WT DMM compared to *Adrb2^-/-^* DMM. **(B)** Body length data are represented as means +/- SEM (n=5 per group). **(C)** Concentration of absolute splenic NE data are represented as means +/- SEM (n=3 per group). Significant p-values are presented as **p ≤ 0.01, when WT DMM compared to *Adrb2^-/-^* DMM. **(D)** Representative images displaying the abdomen of 6 months old unchallenged WT and *Adrb2^-/-^* mice (H - hearth, L - liver, AF – abdominal fat).
