# Supplementary figures and images for: β2-Adrenoceptor Deficiency Results in Increased Calcified Cartilage Thickness and Subchondral Bone Remodeling in Murine Experimental Osteoarthritis
Source: Front Immunol. 2022 Jan 13;12:801505. doi: 10.3389/fimmu.2021.801505 (PMC8794706; doi:10.3389/fimmu.2021.801505)

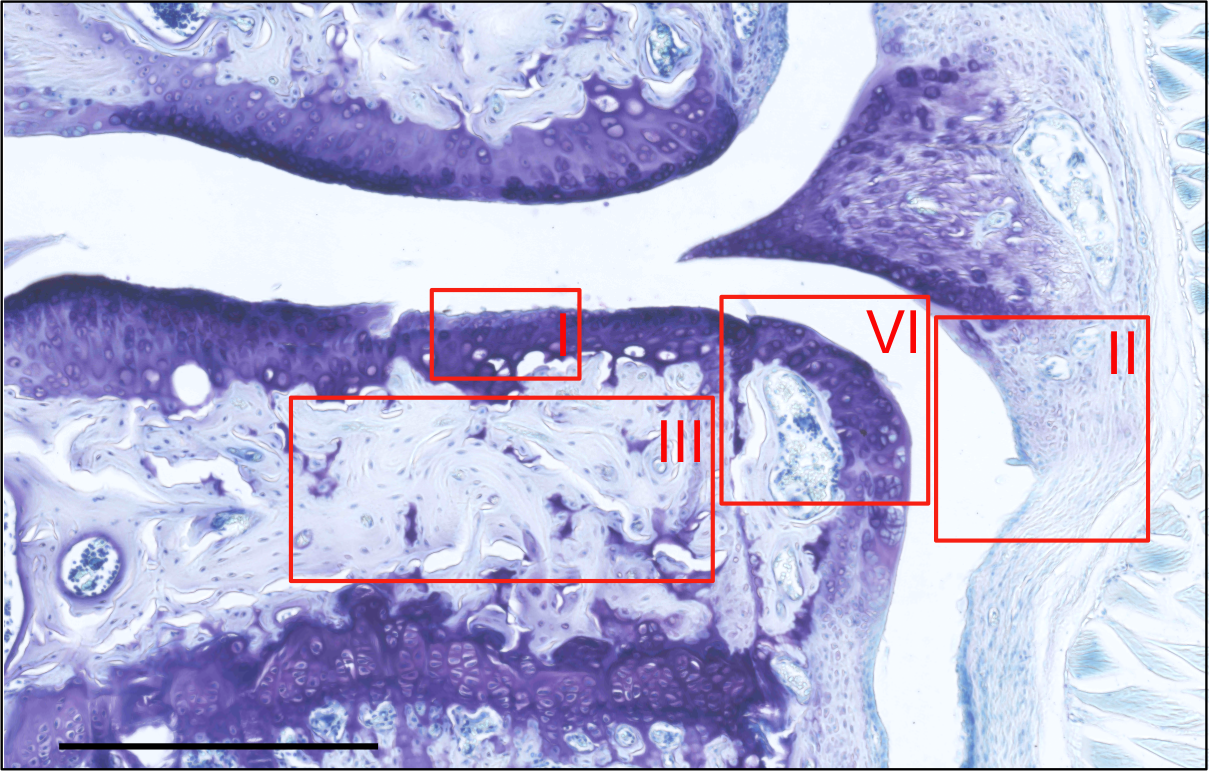

Supplement: Supplementary Figure 1 — Areas of interests in the right murine knee joint. (A) Areas of interest for the analyses indicated by red rectangles for articular cartilage (I), synovium (II), subhcondral bone (III) and region of osteophytes (IV). DMMB staining (bar: 500 µm). [file Image_1.tif]

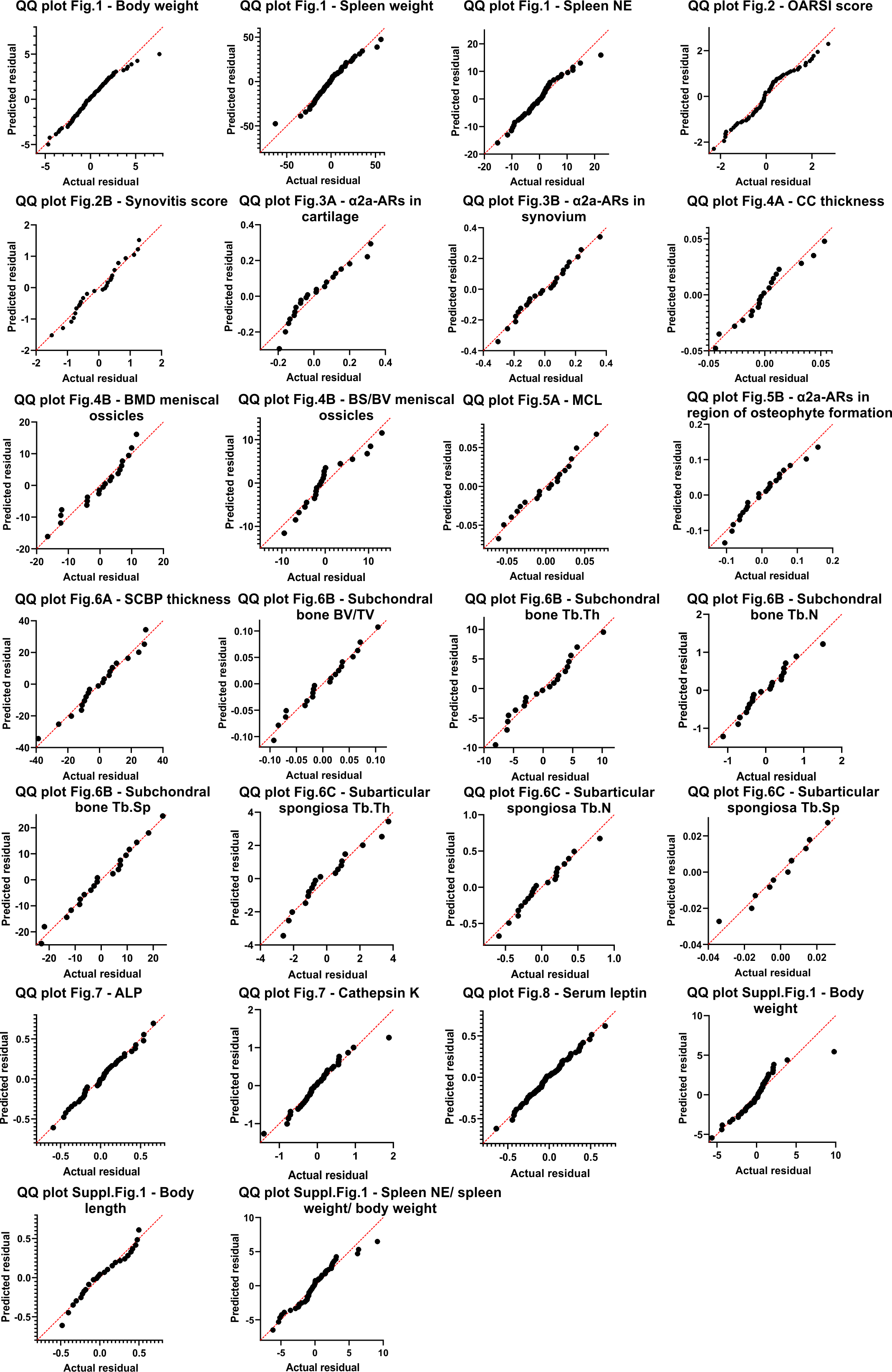

Supplement: Supplementary Figure 2 — Quantile-quantile (QQ) plots of residuals present the theoretical quantiles predicted to check the normal distribution for ANOVA. [file Image_2.tif]

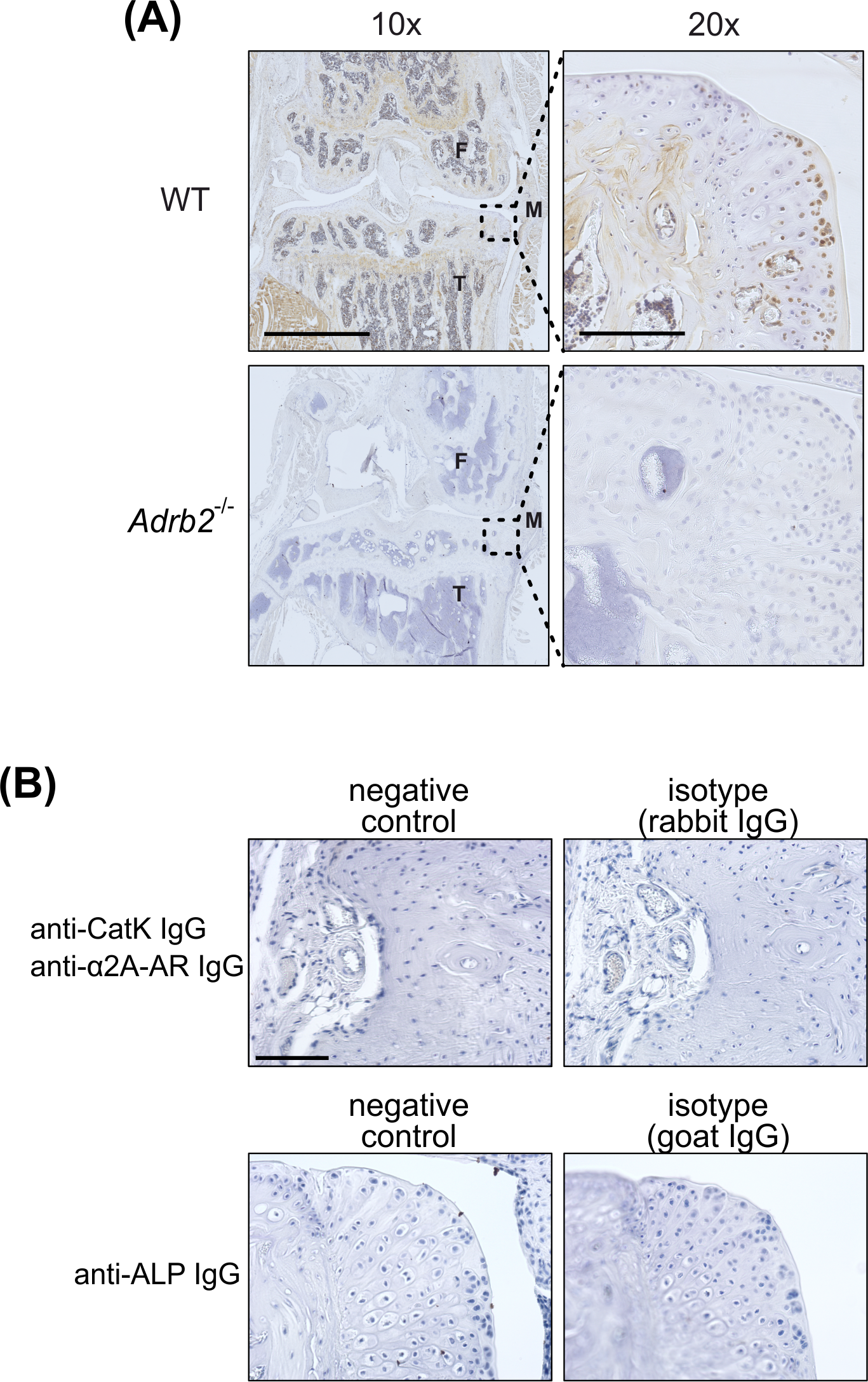

Supplement: Supplementary Figure 3 — Confirmation of β2-AR deficiency and controls for the immunohistochemical stainings (A) Staining of knee joints of β2-AR in WT and Abdrb2 -/- mice with a magnification of 10x for the whole joint (bar: 1,5 mm) and a magnification of 20x for the region of osteophyte formation (bar: 100 µm, F – femur, T – tibia, M - meniscus). (B) Negative and isotype controls for the immunohistochemical stainings in the region of osteophyte formation (bar: 100µm). [file Image_3.tif]

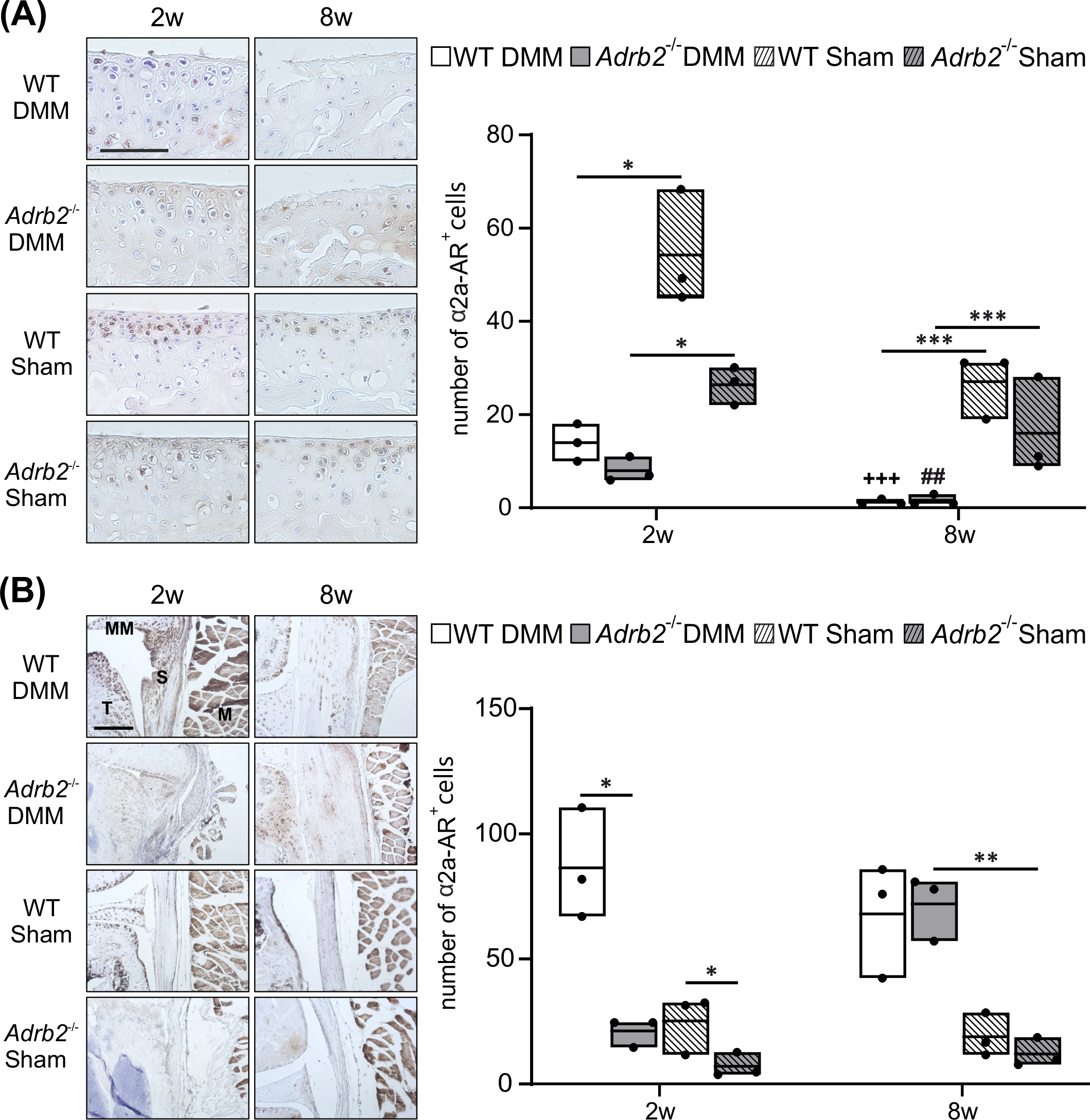

Supplement: Supplementary Figure 4 — AR expression in the articular cartilage and in the synovium. (A) Immunohistochemical detection and quantification of α2A- and β2-AR (dark brown) in the articular cartilage in the medial tibia plateau of WT and Adrb2-/- mice 2 and 8 weeks after DMM or Sham surgery (bar: 100 µm) and (B) in the medial synovium of WT and Adrb2-/- mice 2 and 8 weeks after DMM or Sham surgery (bar: 200 µm) Nuclei are counterstained with hematoxylin (dark blue). Data are presented as box plots with whiskers. Each black circle represents an individual mouse (n=3 per group). Significant p-values are presented as *p ≤ 0.05, **p ≤ 0.01, ***p ≤ 0.001 for comparisons between groups at one time point. +++p ≤ 0.001 when compared to 4 weeks WT DMM; ## p ≤ 0.01 when compared to 4 weeks Adrb2-/- DMM. [file Image_4.tif]

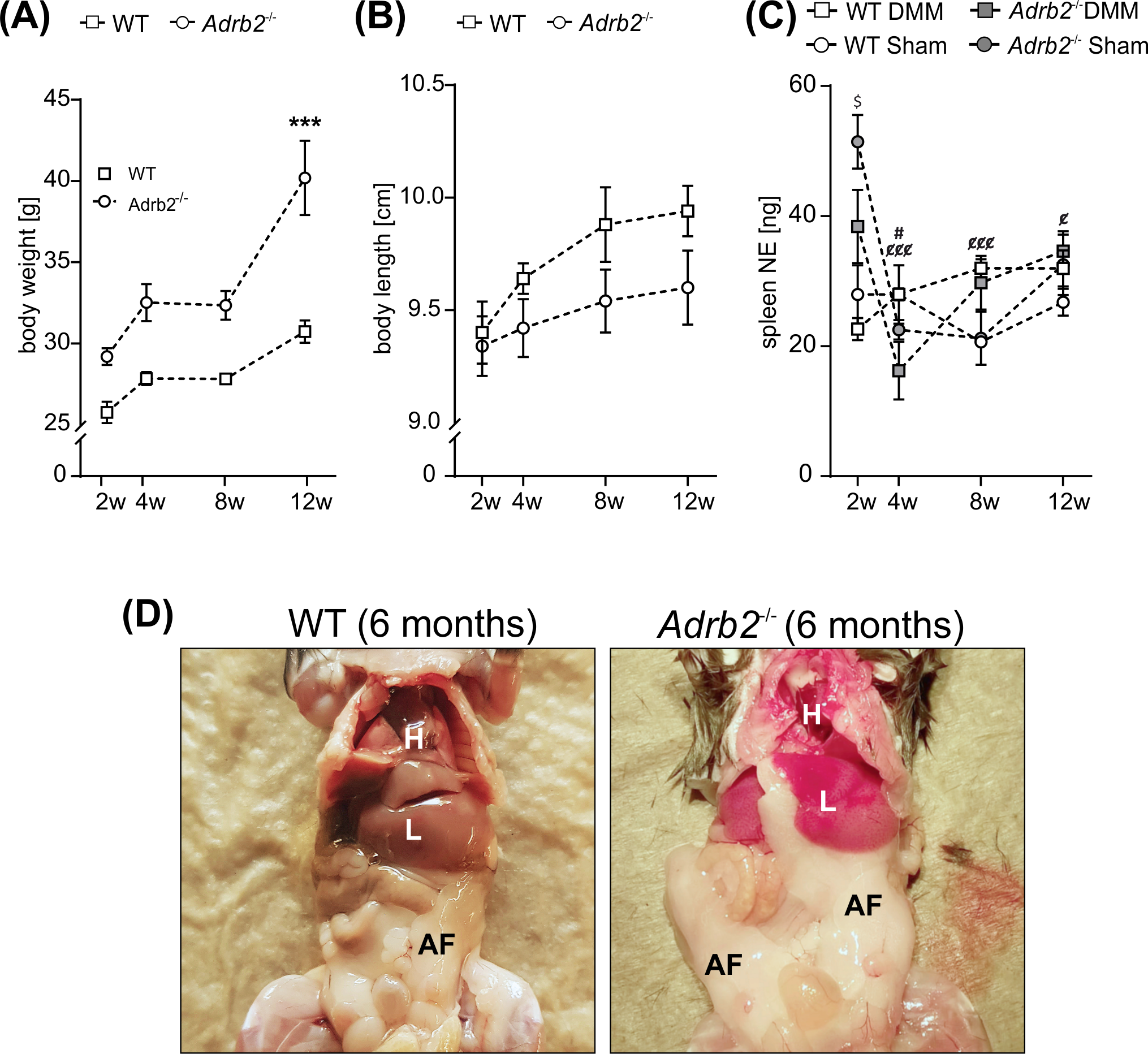

Supplement: Supplementary Figure 5 — Body weight, body length at the same time points in healthy non-operated WT and Adrb2-/- mice like 2, 4, 8 and 12 weeks after DMM or Sham surgery in WT and Adrb2-/- DMM mice. (A) Body weight data are represented as means +/- SEM (n=5 per group). Significant p-values are presented as ***p ≤ 0.001, when WT DMM compared to Adrb2-/- DMM. (B) Body length data are represented as means +/- SEM (n=5 per group). (C) Concentration of absolute splenic NE data are represented as means +/- SEM (n=3 per group). Significant p-values are presented as **p ≤ 0.01, when WT DMM compared to Adrb2-/- DMM. (D) Representative images displaying the abdomen of 6 months old unchallenged WT and Adrb2-/- mice (H - hearth, L - liver, AF – abdominal fat). [file Image_5.tif]
